# Demographic Reconstruction of Antarctic Fur Seals Supports the Krill Surplus Hypothesis

**DOI:** 10.3390/genes13030541

**Published:** 2022-03-18

**Authors:** Joseph I. Hoffman, Rebecca S. Chen, David L. J. Vendrami, Anna J. Paijmans, Kanchon K. Dasmahapatra, Jaume Forcada

**Affiliations:** 1 Department of Animal Behavior, University of Bielefeld, P.O. BOX 100131, 33615 Bielefeld, Germany; rebecca.chen@uni-bielefeld.de (R.S.C.); david.vendrami@edu.unife.it (D.L.J.V.); a.paijmans@uni-bielefeld.de (A.J.P.); 2 British Antarctic Survey, High Cross, Madingley Road, Cambridge CB3 OET, UK; jfor@bas.ac.uk; 3 Department of Biology, University of York, York YO10 5DD, UK; kanchon.dasmahapatra@york.ac.uk

**Keywords:** *Arctocephalus gazella*, Antarctic fur seal, RAD sequencing, demographic modelling, bottleneck, krill surplus hypothesis, marine mammal, baleen whales, pinnipeds

## Abstract

Much debate surrounds the importance of top-down and bottom-up effects in the Southern Ocean, where the harvesting of over two million whales in the mid twentieth century is thought to have produced a massive surplus of Antarctic krill. This excess of krill may have allowed populations of other predators, such as seals and penguins, to increase, a top-down hypothesis known as the ‘krill surplus hypothesis’. However, a lack of pre-whaling population baselines has made it challenging to investigate historical changes in the abundance of the major krill predators in relation to whaling. Therefore, we used reduced representation sequencing and a coalescent-based maximum composite likelihood approach to reconstruct the recent demographic history of the Antarctic fur seal, a pinniped that was hunted to the brink of extinction by 18th and 19th century sealers. In line with the known history of this species, we found support for a demographic model that included a substantial reduction in population size around the time period of sealing. Furthermore, maximum likelihood estimates from this model suggest that the recovered, post-sealing population at South Georgia may have been around two times larger than the pre-sealing population. Our findings lend support to the krill surplus hypothesis and illustrate the potential of genomic approaches to shed light on long-standing questions in population biology.

## 1. Introduction

Anthropogenic exploitation, particularly of ecologically important organisms, can have profound and often unexpected effects on natural ecosystems, influencing community structure, function, and productivity [1,2]. A prime example of this comes from the Southern Ocean, where uncontrolled exploitation of the great whales in the first half of the 20th century [3] resulted in significant ecosystem-level changes [4,5,6,7,8]. Large-scale commercial whaling commenced in the Southern Ocean in 1904 and peaked in 1930, when the annual catch reached almost 40,000 whales [3]. An estimated two million whales were taken between 1904 and 1990, including over 360,000 blue whales, 72,000 fin whales, 400,000 sperm whales, 200,000 humpback whales and 110,000 minke whales [9]. The blue whales alone accounted for around 40 million tonnes of biomass, equivalent to that of a billion people [10].

These hunted whales would have consumed vast amounts of Antarctic krill (*Euphausia superba*), a shrimp-like crustacean that forms the dominant prey of many mammals and birds of the Southern Ocean [8]. Consequently, whaling activities in the Southern Ocean are estimated to have produced a surplus of around 147–380 million tonnes of uneaten krill per year [6,11]. This competitive release of krill is thought to explain increases in populations of seals and penguins at South Georgia during the 1940s to 1970s, a top-down hypothesis referred to as the ‘krill surplus hypothesis’ [6]. However, krill abundance in the Scotia Sea has decreased substantially since the 1970s as the amount of sea ice has declined in response to rising global temperatures [12,13]. Consequently, many krill-dependent Antarctic predators are currently declining as a result of bottom-up changes to the Antarctic marine ecosystem [7,8,14,15].

While arguments have been made for both top-down and bottom-up effects playing important roles in the Southern Ocean ecosystem, their relative importance remains contentious [16,17]. In particular, ecosystem modelling studies have suggested that prey release is sufficient alone to explain 20th century trends in populations of some seals and penguins [4,5] and several empirical observations have lent support to the krill surplus hypothesis [8,18,19,20]. However, some authors have pointed out inconsistencies among species as well as incomplete niche overlap between, for example, resident penguin populations and migratory whale populations [21,22]. Arguably, the greatest barrier to understanding the importance of top-down and bottom-up effects in the Southern Ocean is a lack of pre-whaling population size baseline data [23]. This makes it challenging to evaluate whether the major consumers of krill increased as a consequence of the demise of the whales, which is important for understanding how these predators might respond to the subsequent recovery of certain whale species [16].

A compelling approach to circumvent this lack of historical baselines is to reconstruct recent population size changes of krill predators from molecular genetic data. This approach has so far only been attempted by a single study [23], which estimated the long-term coalescent effective population size (*N*_e_) of the Antarctic minke whale from eleven nuclear markers and converted the resulting value into a census population size estimate. This fell within the range of several contemporary abundance estimates and was interpreted as meaning that the number of Antarctic minke whales was not unusually high after whaling. However, this study was criticised [24] because effective and census population sizes are not directly comparable, with *N*_e_ usually being much smaller [25], and because molecular diversity (θ) based estimates of the long-term coalescent *N*_e_ integrate information over long timeframes (in the order of 4*N*_e_ generations), so are unlikely to be strongly affected by recent anthropogenic perturbations [26].

Alternatively, changes in *N*_e_ can be inferred from the site frequency spectrum (SFS). The SFS is the distribution of allele frequencies of a given set of loci within a population [27] and ranges from rare ‘singletons’, in which an allele appears only once in the sample of individuals, to high frequency alleles that are carried by the majority of individuals. Importantly, the SFS is directly affected by a population’s demographic history; bottlenecks decrease the number of rare alleles [28], whereas population expansions lead to an excess [29,30]. Various methods have been developed for inferring demographic histories from the SFS [31,32,33,34] including the program fastsimcoal2, which uses a composite likelihood based framework to robustly infer demographic parameters under complex demographic scenarios [35]. Estimating the SFS requires high-resolution data from multiple individuals, so SFS-based demographic inference was historically limited to species for which large genomic datasets were available [36,37]. However, the emergence of reduced representation sequencing approaches such as restriction site associated DNA (RAD) sequencing [38] has meant that population genomic data can now be generated at reasonable cost for practically any species. Consequently, increasing numbers of studies are implementing SFS-based demographic reconstruction in wild populations [39,40,41,42].

The Antarctic fur seal (*Arctocephalus gazella*) is ideally suited to testing the krill surplus hypothesis using a population genomic approach. This polygynous and site-faithful pinniped [43,44,45,46] breeds on sub-Antarctic islands, with around 97% of the global population being concentrated around South Georgia [47]. Commercial sealing began shortly after the discovery of South Georgia by Captain James Cook in 1775 and continued unabated until the 1820s, by which point around 1.2 million Antarctic fur seals are believed to have been taken [48]. After the last commercial catch of 170 individuals at South Georgia in 1908, the species was considered all but extinct. For several decades, hardly any individuals were sighted ashore [49] until the Discovery expedition of 1936, when a small breeding population with 12 pups was observed at South Georgia [47]. This was followed by a period of explosive population growth during the 1960s and 1970s [50], which culminated in the South Georgia population reaching an estimated 2.7 million individuals in 1990 [47] and potentially as many as four to seven million individuals by the late 1990s [51]. However, it is difficult to gather reliable census data when population sizes are very large [51] and there is also some evidence to suggest that the 1990 census may have been conducted during an atypical breeding season [47]. Furthermore, the larger estimate [51] is based on data from an single breeding colony on Bird Island and it is unclear to what extent these data can be extrapolated to the whole of South Georgia.

**Figure 1:**
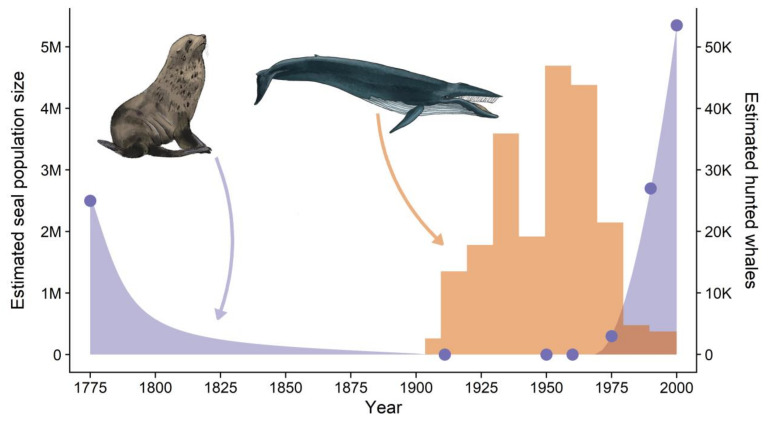
Graphical representation of temporal trends in Antarctic fur seal abundance and baleen whale harvesting. The Antarctic fur seal was heavily hunted in the late 18th and early 19th centuries and was considered virtually extinct by the early 1900s. Subsequently, explosive population growth during the second half of the 20th century coincided with the harvesting of the baleen whales [5]. Temporal changes in Antarctic fur seal abundance are reconstructed from empirical population size estimates from the scientific literature (purple points, [47,49,51,55,56,57]). Numbers of harvested baleen whales originate from [58]. Comparable whale census population size estimates from the same period are not available. Similarly, although data are available on the annual numbers of sealing vessels that visited South Georgia [59], the number of seals taken was often not recorded, meaning that it is not possible to depict temporal changes in the number of harvested seals. Original artwork by Elena Fissenewert.

Despite these uncertainties, the rapid mid-20th century growth of the Antarctic fur seal population at South Georgia has been described as ‘unprecedented in pinnipeds’ [52]. Temporal concordance between this explosive growth phase and the harvesting of the whales (Figure 1) is therefore considered a key piece of evidence supporting the krill surplus hypothesis [5]. Accordingly, it has been speculated that this release of krill increased the carrying capacity of the environment for Antarctic fur seals and allowed the post-sealing population at South Georgia to exceed its historical abundance [53,54]. However, census data from the pre-sealing population are lacking and the only available historical estimate of 2.5 million individuals, based on a reconstruction of the number of harvested pelts and a female-only, age-structured population model, has a very high level of associated uncertainty (95% CI = 1.5–3.5 million individuals) [55].

Here, we used RAD sequencing and a coalescent-based maximum composite likelihood approach to reconstruct the recent demographic history of the Antarctic fur seal. Specifically, we used the empirical SFS together with coalescent simulations in fastsimcoal2 to estimate the likelihood of our data under two alternative demographic scenarios, one incorporating a severe reduction in *N*_e_ during the timeframe when sealing is known to have taken place (the ‘bottleneck model’), and the other assuming no recent changes in *N*_e_ (the ‘null model’). The best supported model was then used to estimate relevant parameters including *N*_e_ values before, during, and after the bottleneck. We hypothesised that Antarctic fur seals would show a genome-wide signature of a strong demographic reduction caused by sealing. In line with the krill surplus hypothesis, we further hypothesised that competitive prey release due to the harvesting of the whales may have facilitated the demographic recovery of the South Georgia population and allowed it to attain a larger size than was present before sealing.

## 2. Materials and Methods

### 2.1. RAD Sequencing Data

RAD sequencing data were generated for 70 Antarctic fur seal individuals sampled during 1996–2001 from Bird Island, South Georgia, as described by Humble et al. [60]. Our dataset includes previously published data for 57 individuals [60] plus unpublished data for a further 13 individuals. Briefly, skin plugs were collected in the field using piglet ear notching pliers and whole genomic DNA was extracted using a modified phenol-chloroform protocol [61]. RAD libraries were prepared following Etter et al. [62], with minor modifications as described in Humble et al. [60], and were 250 bp paired-end sequenced on an Illumina Hiseq 1500 (Hayward, CA, USA).

### 2.2. Bioinformatic Data Processing

Read quality was assessed using FastQC v0.11.9 and sequences were trimmed to 225 bp and demultiplexed using process_radtags in STACKS v1.41 [63]. The reads were then mapped to the Antarctic fur seal reference genome v1.4 [64] using BWA MEM v0.7.13 [65] with default parameters. The resulting SAM files were converted to BAM format, sorted by coordinates and indexed using SAMtools v1.11 [66]. The BAM files were then used as input for calculating genotype likelihoods and the SFS using -doSaf and -realSFS in ANGSD v0.935 [67]. Due to the absence of ancestral state information, we estimated the folded SFS. Individual genotype likelihoods were calculated assuming Hardy–Weinberg equilibrium using the algorithm provided by SAMtools (-doSaf 1, -GL 1). Only sites with a minimum mapping quality of 20 and a minimum qscore of 20 were included (-minMapQ 20, -minQ 20). The qscores around indels were adjusted and bad reads were discarded (-baq 1, -remove-bads 1). We only retained sites that were present in a minimum of 90% of all individuals with a minimum depth of coverage of five and maximum depth of coverage of 58 per individual (-minInd 63, -setMinDepth 315, -setMaxDepth 3600). To estimate the SFS using the -realSFS command, the maximum number of iterations of the EM algorithm was set to 1000.

### 2.3. Demographic Modelling

We first simulated SFS based on two alternative demographic scenarios. The bottleneck model simulated a reduction in *N*_e_ followed by exponential population growth. For this model, we estimated the pre-sealing effective population size (*N*_e_pre-sealing), the bottleneck effective population size (*N*_e_bot), the growth rate after the bottleneck (GR) and the post-sealing effective population size (*N*_e_post-sealing). By contrast, the null model assumed no population growth or decline and estimated only *N*_e_post-sealing. In both models, the defined initial search ranges for *N*_e_post-sealing were log uniformly distributed between 5000 and 50,000 diploid individuals. For the bottleneck model, the defined initial search range for *N*_e_pre-sealing was uniformly distributed between 5000 and 50,000 individuals, while the defined initial search range for *N*_e_bot was uniformly distributed between 10 and 250 individuals. However, the composite maximum likelihood approach implemented in fastsimcoal2 uses these search ranges solely as starting values and parameter values can therefore exceed the upper limits [35]. Detailed sealing records [59] were used to simplify the bottleneck model by fixing the timing of the start and end of the bottleneck to 22 and 11 generations ago, respectively, assuming a generation time of ten years [68]. For both models, we simulated data under a fixed mutation rate of 2.5 × 10^−8^, in line with marine mammal mutation rate estimates from the literature [69]. The growth rate in the bottleneck model was defined as a complex parameter log (*N*_e_bot/*N*_e_post-sealing)/11. A total of 100 replicate runs were performed for each model, including 100 estimation loops with 100,000 coalescent simulations. We did not include singletons in the simulations, as these have been found to be biased when sequence coverage is low [70]. Out of the 100 replicates for each model, the run with the highest maximum likelihood was retained. The best model was then determined based on the delta likelihood values (difference between the estimated and observed likelihoods) and Akaike’s information criterion (AIC) = 2 * ln(likelihood) + 2 * *K* where ln is the natural logarithm, likelihood is the maximum likelihood, and *K* is the number of parameters in the model. Moreover, to evaluate the fit of the best model to our data, we simulated 600 SFS based on the parameter estimates from the best model and visually compared the resulting SFS with the observed SFS.

Finally, we investigated the uncertainty of our parameter estimates using a non-parametric bootstrap approach. Specifically, we used ANGSD to bootstrap the SFS and to generate 600 SFS estimates based on data that were subsampled with replacement. This was implemented using the option-bootstrap within the ‘realSFS’ module of ANGSD. Then, for each of these 600 SFS, the parameters for the model were re-estimated based on 100 replicate runs, each including 100 estimation loops with 100,000 coalescent simulations. For each SFS, the run with the top maximum likelihood was retained and used for the bootstrap distribution. For each parameter, 95% confidence intervals (CIs) were then calculated based on the resulting 600 bootstrap estimates.

## 3. Results

**Figure 2:**
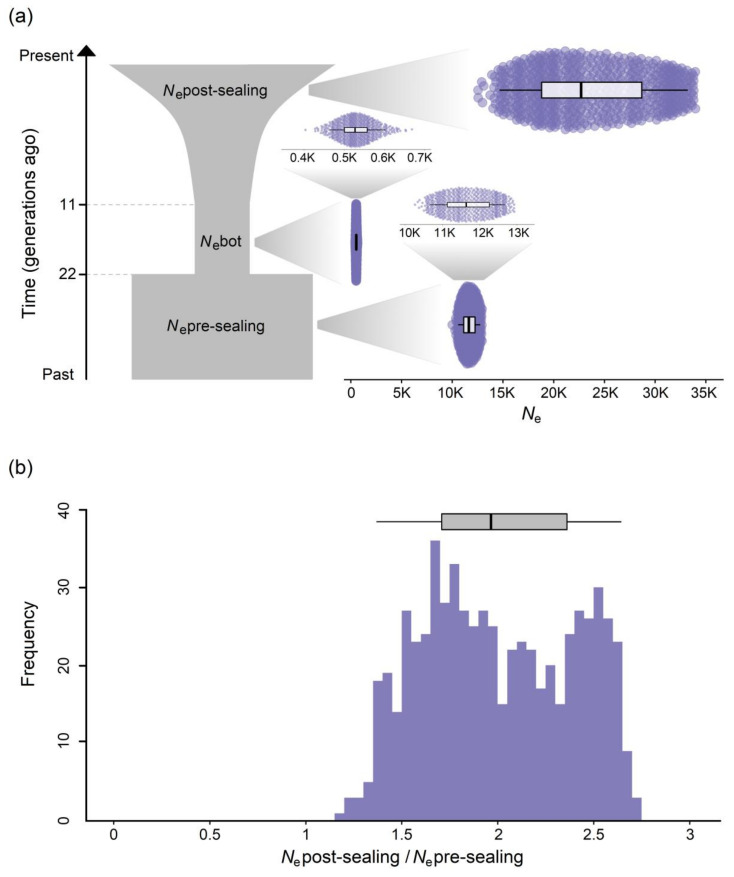
Reconstruction of the recent demographic history of the Antarctic fur seal based on population genomic data. (**a**) Schematic of the best supported demographic model showing the estimated parameter values for *N*_e_pre-sealing, *N*_e_bot, and *N*_e_post-sealing. Dashed grey lines represent the fixed values for the start and end of the bottleneck. The priors for the estimated parameters are described in the Materials and methods. For all three parameters, distributions of 600 non-parametric bootstrap estimates are plotted together with box plots showing median values, and 25th and 75th percentiles, with the whiskers representing 95% confidence intervals. The *N*_e_ estimates are plotted on the same scale along the *x*-axis for comparability, while the distributions of *N*_e_pre-sealing and *N*_e_bot can be seen in the zoom-ins. (**b**) Distribution of the ratio of *N*_e_post-sealing to *N*_e_pre-sealing over all 600 bootstrapped datasets. The box plot above the bar chart shows the median value, and 25th and 75th percentiles, with the whiskers representing the 95% confidence interval.

RAD sequencing produced an average of 1,409,505 (range = 328,537–4,488,652) 250 bp paired-end reads per individual. For each individual, a mean of 99.4% of the reads were aligned to the reference genome (range = 95.9–99.8%). A total of 31,328,941 positions with estimated genotype likelihoods were used to estimate the SFS, of which 1.2% were polymorphic. In line with the known demographic history of the Antarctic fur seal, the bottleneck model achieved the highest support, having both a higher maximum likelihood and a lower AIC than the null model (Table 1). Furthermore, simulated SFS based on the best supported model showed a similar distribution to the observed SFS (Supplementary Figure S1), indicating that this model provides a good fit to the empirical data. Based on this model, *N*_e_pre-sealing, *N*_e_bot, and *N*_e_post-sealing were estimated at 12,506, 534, and 29,319, respectively (Table 1). To investigate model uncertainty, we used a non-parametric bootstrapping approach to produce 600 site frequency spectra by subsampling the empirical SFS with replacement. Although there was some variation in the uncertainty of the estimated parameter values (95% CIs: *N*_e_pre-sealing = 10,589–12,760, *N*_e_bot = 452–615 and *N*_e_post-sealing = 14,667–33,222), none of the 95% CIs overlapped (Figure 2a). Furthermore, the ratio of *N*_e_post-sealing to *N*_e_pre-sealing averaged 2.0 (95% CI: 1.37–2.64) across all 600 simulations and was always greater than one (Figure 2b).

## 4. Discussion

We used RAD sequencing to infer recent changes in the size of the Antarctic fur seal population at South Georgia. In line with expectations based on known history of this species, our best supported model incorporated a recent bottleneck followed by exponential population growth. Furthermore, maximum likelihood estimates from this model suggest that the post-sealing *N*_e_ may have been around twice as large as the pre-sealing *N*_e_. Our study builds upon ecosystem modelling [4,5] and empirical studies of multiple krill consumers [8,18,19,20] that lend support to the krill surplus hypothesis.

### 4.1. Bottleneck Inference

Our best supported model included a substantial demographic reduction during the known time period of commercial sealing. However, the maximum likelihood estimate of *N*_e_bot was both larger (534 versus ~150–300) and more precise than previous estimates based on microsatellites [59,71,72]. This probably reflects differences in both the genetic markers themselves and the analytical approach. One key methodological difference is that previous studies used approximate Bayesian computation, which produces estimates that are constrained by pre-defined priors, whereas fastsimcoal uses search ranges as starting values but the resulting parameter estimates can exceed the upper limits [35]. Accordingly, an exploratory analysis by Hoffman et al. [59] produced a more comparable *N*_e_bot estimate of around 700 when the priors for this parameter were relaxed.

An *N*_e_bot of 534 equates to a census population size substantially larger than Larsen’s [49] estimate of 30 individuals at South Georgia in 1911. Furthermore, mammalian effective population sizes are usually several times smaller than census population sizes [25], implying that thousands of animals may have escaped sealing at South Georgia. This is consistent with previous observations of high genetic diversity [71] and rapidly decaying linkage disequilibrium across the genome [60] in the South Georgia population. Furthermore, recent studies have uncovered a strong population genetic structure across the species’ circumpolar range [60,72,73,74], implying that at least four refugial populations survived commercial sealing. Thus, it appears that the species as a whole may have been more resilient to commercial exploitation than was previously believed, probably because small remnants of historically much larger populations were able to persist in a handful of remote and inaccessible locations [75].

### 4.2. Pre- and Post-Sealing Effective Population Sizes

A lack of pre-whaling baseline data on seal and penguin populations has made it challenging to evaluate the ecosystem-level consequences of historical whaling [23]. We circumvented this issue by reconstructing temporal changes in Antarctic fur seal abundance from RAD sequencing data. This approach has the advantage of producing *N*_e_ estimates that are directly comparable between different time points. In contrast to previous attempts at demographic reconstruction using microsatellites [59,71,72], we were able to precisely estimate *N*_e_pre-sealing, as indicated by the relatively narrow range of parameter estimates resulting from the non-parametric bootstrapping (95% CI = 10,589–12,760). Our estimate of the post-sealing *N*_e_ was somewhat less precise, with the 95% CI ranging from 14,667 to 33,222. This is probably because the population boom was short lived, lasting for only a few generations, which would not have been long enough to fully erase the imprint of the long-term *N*_e_ on the SFS. Nevertheless, the 95% CIs of *N*_e_pre-sealing and *N*_e_post-sealing did not overlap and the ratio of *N*_e_post-sealing to *N*_e_pre-sealing was consistently greater than one across all 600 bootstrapped datasets. Consequently, although there is some uncertainty associated with our parameter estimates, statistically speaking, there is a low probability that *N*_e_post-sealing and *N*_e_pre-sealing are the same.

We also recognize that our *N*_e_ estimates are small in comparison to available census size estimates, which run into the millions of individuals (e.g., [47,51]). However, this is to be expected given that Antarctic fur seals exhibit strong polygyny [43,46], natal site fidelity [45], and population structure [60,72,73], all traits that are expected to reduce the ratio of the effective to census population size. Accordingly, our *N*_e_ estimates are comparable to other estimates from the literature [76] including genomic estimates of the long-term coalescent *N*_e_ from several pinniped species [26].

Some authors have argued that competitive prey release may have allowed the Antarctic fur seal population at South Georgia to increase beyond its pre-exploitation size [53,54]. However, these arguments were largely motivated by anecdotal observations, such as heavy damage to the tussock grass in the mid-1980s, which Bonner [53] described as ‘a new phenomenon associated with high density occupation by the recovering population’. While these kinds of observations can be prone to confirmation bias, our results suggest that the post-sealing population at South Georgia may indeed have been larger than the pre-sealing population. A similar conclusion was also reached by the authors of a paleolimnological study at Signy Island, which found over four times more Antarctic fur seal hairs in lake sediments from the 1990s than at any point during the past 6000 years [18]. While both of these studies ostensibly support the krill hypothesis, it is important to recognize that the density of Antarctic krill had already begun to decline before the fur seal population reached its peak in the 1990s [12]. One possible explanation for this lag [5] could be that the biomass of krill at South Georgia was sufficient to support the growing fur seal population until the 1990s, by which point the krill surplus would have come to an end (see below).

### 4.3. The Role of Bottom-Up Effects

Although it has been argued that the krill surplus hypothesis provides a valid explanation for increases in populations of penguins and seals during the mid-20th century [4], it cannot account for more recent declines of many Antarctic predators [4,8,10,14,22]. In practice, the excess krill biomass appears to have been eroded during the last quarter of the 20th century by a decline in the primary productivity of the Southern Ocean [4]. This has been linked to a reduction in the bioavailability of whale-recycled iron [4,10,11], as well as to climate-driven declines in sea ice extent and krill density in the Western Antarctic Peninsula and Scotia Sea [8,12,13,16]. In the case of the Antarctic fur seal, the number of females breeding at a long-term study population on Bird Island shows tight linkage to local krill availability [14] and there has also been a recent switch from positive to negative dependent pup mortality [77]. Consequently, large-scale changes in krill biomass may help to explain both the rapid post-sealing recovery of the fur seal population and its subsequent, ongoing decline.

### 4.4. Caveats

Although our study illustrates the utility of coalescent based simulation for inferring recent demographic histories, several important limitations are associated with this approach. First, computation of the SFS from genomic data can be challenging, particularly for low coverage data where inaccurate genotype calls can lead to biased estimation of the SFS [78,79,80]. To compensate for this, we implemented a genotype likelihood approach that incorporates uncertainty due to sequencing errors, variation in the depth of coverage and alignment quality [67]. Additionally, we excluded singletons from our analysis as these can be especially error prone in low coverage datasets [81]. Finally, non-parametric bootstrapping of the SFS allowed us to quantify the uncertainty associated with our maximum likelihood estimates. This was generally quite low, suggesting that our results are reasonably robust.

Second, alternative demographic trajectories can potentially produce the same SFS in a single population [82]. This is a limitation inherent to all demographic inference approaches in which demographic scenarios must be pre-specified. However, the recent history of the Antarctic fur seal is extraordinarily well documented [48,59,83,84] so we believe that our bottleneck model is a good representation of the true demographic history both in terms of the time parameters and population size priors. However, we cannot exclude the possibility that an alternative (although arguably less parsimonious) scenario might fit the data equally well.

Third, our study focused on a single species out of a suite of Southern Ocean predators. Thus, although our results are consistent with the krill surplus hypothesis, they do not allow us to infer causality. In the future, it would be interesting to conduct parallel analyses of SFS from multiple Southern Ocean predators including seals, penguins and smaller whale species. Although this approach would entail significant challenges in terms of data collection, it would allow the investigation of competitive release via the analysis of concurrent demographic patterns across multiple coexisting predators.

## 5. Conclusions

We used RAD sequencing to reconstruct the recent demographic history of the Antarctic fur seal population at South Georgia. We found evidence of a severe demographic reduction due to commercial sealing. Furthermore, maximum likelihood estimates of the pre- and post-sealing *N*_e_ from our best supported model were consistent with independent observations suggesting that prey release may have facilitated a rapid demographic increase in the recovering Antarctic fur seal population at South Georgia. Our results build upon previous modelling and empirical studies supporting the krill surplus hypothesis, although ongoing climate change has been linked to more recent declines of many Southern Ocean predators including Antarctic fur seals.

## Figures and Tables

**Table 1:** Relative likelihoods of the two alternative demographic models together with AIC values and parameter estimates for the pre-sealing effective population size (*N*_e_pre-sealing), the bottleneck effective population size (*N*_e_bot), and the post-sealing effective population size (*N*_e_post-sealing). See the Materials and methods for details.

Model	Max(log_10_(likelihood)) ^a^	Number of Parameters	AIC	*N*_e_pre-Sealing	*N*_e_bot	*N*_e_post-Sealing
Bottleneck model	−882,361.6	3	4,063,431	12,506	534	29,319
Null model	−882,378.8	1	4,063,506	–	–	14,789

^a^ Based on the best likelihood among the 100 independent runs for each model.

## Data Availability

The raw RAD sequencing reads are available at the short-read archive (SRA) of the National Biotechnology Centre of Information (NCBI) under BioProject ID PRJNA473050, SRA accession SRP148937. All codes used for our data analyses can be found in our GitHub repository: https://github.com/rshuhuachen/furseal-demography (last accessed on 14 March 2022).

## Supplementary Materials

The following supporting information can be downloaded at https://www.mdpi.com/article/10.3390/genes13030541/s1: Figure S1, Observed and simulated site frequency spectra (SFS).

## References

[1] Enquist B.J., Abraham A.J., Harfoot M.B.J., Malhi Y., Doughty C.E. (2020). The megabiota are disproportionately important for biosphere functioning. Nat. Commun..

[2] Doughty C.E., Roman J., Faurby S., Wolf A., Haque A., Bakker E.S., Malhi Y., Dunning J.B., Svenning J.-C. (2016). Global nutrient transport in a world of giants. Proc. Natl. Acad. Sci. USA.

[3] Hofman R.J. (2017). Sealing, whaling and krill fishing in the Southern Ocean: Past and possible future effects on catch regulations. Polar Rec..

[4] Surma S., Pakhomov E.A., Pitcher T.J. (2014). Effects of Whaling on the Structure of the Southern Ocean Food Web: Insights on the “Krill Surplus” from Ecosystem Modelling. PLoS ONE.

[5] Mori M., Butterworth D.S. (2006). A first step towards modelling the krill-predator dynamics of the Antarctic ecosystem. CCAMLR Sci..

[6] Laws R.M. (1977). Seals and whales of the Southern Ocean. Philosphical Trans. R. Soc. Lond. B.

[7] Trathan P.N., Ratcliffe N., Masden E.A. (2012). Ecological drivers of change at South Georgia: The krill surplus, or climate variability. Ecography.

[8] Trivelpiece W.Z., Hinke J.T., Miller A.K., Reiss C.S., Trivelpiece S.G., Watters G.M. (2011). Variability in krill biomass links harvesting and climate warming to penguin population changes in Antarctica. Proc. Natl. Acad. Sci. USA.

[9] Rocha C.R., Clapham P., Ivashchenko Y. (2015). Emptying the oceans: A summary of industrial whaling catches in the 20th century. Mar. Fish. Rev..

[10] Smetacek V., Durarte C.M. (2008). Are declining Antarctic krill stocks a result of global warming or decimation of the whales?. Invited Lecture, Scientific Debate on Impacts of Global Warming on Polar Ecosystems.

[11] Savoca M.S., Czapanskiy M.F., Kahane-Rapport S.R., Gough W.T., Fahlbusch J.A., Bierlich K.C., Segre P.S., Di Clemente J., Penry G.S., Wiley D.N. (2021). Baleen whale prey consumption based on high-resolution foraging measurements. Nature.

[12] Atkinson A., Siegel V., Pakhomov E., Rothery P. (2004). Long-term decline in krill stock and increase in salps within the Southern Ocean. Nature.

[13] Atkinson A., Siegel V., Pakhomov E., Rothery P., Loeb V., Ross R., Quetin L., Schmidt K., Fretwell P., Murphy E. (2008). Oceanic circumpolar habitats of Antarctic krill. Mar. Ecol. Prog. Ser..

[14] Forcada J., Hoffman J.I. (2014). Climate change selects for heterozygosity in a declining fur seal population. Nature.

[15] Croxall J.P., Trathan P.N., Murphy E.J. (2002). Environmmental change and Antarctic seabird populations. Science.

[16] Nicol S., Croxall J.P., Trathan P., Gales N., Murphy E. (2007). Paradigm misplaced? Antarctic marine ecosystems are affected by climate change as well as biological processes and harvesting. Antarct. Sci..

[17] Ainley D., Ballard G., Ackley S., Blight L.K., Eastman J.T., Emslie S.D., Lescroel A., Olmastroni S., Townsend S.E., Tynan C.T. (2007). Paradigm lost, or is top-down forcing no longer significant in the Antarctic marine ecosystem?. Antarct. Sci..

[18] Hodgson D.A., Johnston N.M., Caulkett A.P., Jones V.J. (1998). Palaeolimnology of Antarctic fur seal *Arctocephalus gazella* populations and implications for Antarctic management. Biol. Conserv..

[19] Emslie S.D., Polito M.J., Patterson W.P. (2013). Stable isotope analysis of ancient and modern gentoo penguin egg membrane and the krill surplus hypothesis in Antarctica. Antarct. Sci..

[20] Emslie S.D., Patterson W.P. (2007). Abrupt recent shift in 13C and 15N values in Adelie penguin eggshell in Antarctica. Proc. Natl. Acad. Sci. USA.

[21] Croxall J.P. (1992). Southern ocean environmental changes: Effects on seabird, seal and whale populations. Philosphical Trans. R. Soc. Lond. B.

[22] Fraser W.R., Trivelpiece W.Z., Ainley D.G., Trivelpiece S.G. (1992). Increases in Antarctic penguin populations: Reduced competition with whales or a loss of sea ice due to environmental warming?. Polar Biol..

[23] Ruegg K.C., Anderson E.C., Scott Baker C., Vant M., Jackson J.A., Palumbi S.R. (2010). Are Antarctic minke whales unusually abundant because of 20th century whaling?. Mol. Ecol..

[24] The Institute Of Cetacean Research ICR Comments on the Paper ‘Are Antarctic Minke Whales Uniusually Abundant Because of 20th Century Whaling?. https://www.icrwhale.org/pdf/100120Release.pdf.

[25] Frankham R. (1995). Effective population size/adult population size ratios in wildlife: A review. Genet. Res..

[26] Peart C.R., Tusso S., Pophaly S.D., Botero-Castro F., Wu C.C., Aurioles-Gamboa D., Baird A.B., Bickham J.W., Forcada J., Galimberti F. (2020). Determinants of genetic variation across eco-evolutionary scales in pinnipeds. Nat. Ecol. Evol..

[27] Nielsen R. (2000). Estimation of population parameters and recombination rates from single nucleotide polymorphisms. Genetics.

[28] Nei M., Maruyama T., Chakraborty R. (1975). The Bottleneck Effect and Genetic Variability in Populations. Evolution.

[29] Tajima F. (1989). The effect of change in population size on DNA polymorphism. Genetics.

[30] Slatkin M., Hudson R.R. (1991). Pairwise comparisons of mitochondrial DNA sequences in stable and exponentially growing populations. Genetics.

[31] Marth G.T., Czabarka E., Murvai J., Sherry S.T. (2004). The Allele Frequency Spectrum in Genome-Wide Human Variation Data Reveals Signals of Differential Demographic History in Three Large World Populations. Genetics.

[32] Adams A.M., Hudson R.R. (2004). Maximum-Likelihood Estimation of Demographic Parameters Using the Frequency Spectrum of Unlinked Single-Nucleotide Polymorphisms. Genetics.

[33] Gutenkunst R.N., Hernandez R.D., Williamson S.H., Bustamante C.D. (2009). Inferring the joint demographic history of multiple populations from multidimensional SNP frequency data. PLoS Genet..

[34] Chen H. (2012). The joint allele frequency spectrum of multiple populations: A coalescent theory approach. Theor. Popul. Biol..

[35] Excoffier L., Dupanloup I., Huerta-Sanchez E., Sousa V.C., Foll M. (2013). Robust demographic inference from genomic and SNP data. PLoS Genet..

[36] Malaspinas A.-S., Westaway M.C., Muller C., Sousa V.C., Lao O., Alves I., Bergström A., Athanasiadis G., Cheng J.Y., Crawford J.E. (2016). A genomic history of Aboriginal Australia. Nature.

[37] Batini C., Hallast P., Zadik D., Delser P.M., Benazzo A., Ghirotto S., Arroyo-Pardo E., Cavalleri G.L., De Knijff P., Dupuy B.M. (2015). Large-scale recent expansion of European patrilineages shown by population resequencing. Nat. Commun..

[38] Baird N.A., Etter P.D., Atwood T.S., Currey M.C., Shiver A.L., Lewis Z.A., Selker E.U., Cresco W.A., Johnson E.A. (2008). Rapid SNP discovery and genetic mapping using sequenced RAD markers. PLoS ONE.

[39] Roesti M., Kueng B., Moser D., Berner D. (2015). The genomics of ecological vicariance in threespine stickleback fish. Nat. Commun..

[40] Pujolar J.M., Dalén L., Hansen M.M., Madsen J. (2017). Demographic inference from whole-genome and RAD sequencing data suggests alternating human impacts on goose populations since the last ice age. Mol. Ecol..

[41] Nunziata S.O., Lance S.L., Scott D.E., Lemmon E.M., Weisrock D.W. (2017). Genomic data detect corresponding signatures of population size change on an ecological time scale in two salamander species. Mol. Ecol..

[42] Pedersen C.-E.T., Albrechtsen A., Etter P.D., Johnson E.A., Orlando L., Chikhi L., Siegismund H.R., Heller R. (2018). A southern African origin and cryptic structure in the highly mobile plains zebra. Nat. Ecol. Evol..

[43] Hoffman J.I., Boyd I.L., Amos W. (2003). Male reproductive strategy and the importance of maternal status in the Antarctic fur seal *Arctocephalus gazella*. Evolution.

[44] Hoffman J.I., Trathan P.N., Amos W. (2006). Genetic tagging reveals extreme site fidelity in territorial male Antarctic fur seals Arctocephalus gazella. Mol. Ecol..

[45] Hoffman J.I., Forcada J. (2012). Extreme natal philopatry in female Antarctic fur seals (*Arctocephalus gazella*). Mamm. Biol..

[46] Bonin C.A., Goebel M.E., Hoffman J.I., Burton R.S. (2014). High male reproductive skew in a low density Antarctic fur seal (*Arctocephalus gazella*) breeding colony. Behav. Ecol..

[47] Boyd I.L. (1993). Pup production and distribution of breeding Antarctic fur seals (*Arctocephalus gazella*) at South Georgia. Antarct. Sci..

[48] Weddell J. (1827). A Voyage Towards the South Pole Performed in the Years 1822–1824.

[49] Larsen C.A. (1920). Report of the Interdepartmental Committee on Research and Development in the Dependencies of the Falklands Islands.

[50] Payne M.R. (1997). Growth of a fur seal population. Philos. Trans. R. Soc. London. B Biol. Sci..

[51] Scientific Committee on Antarctic Research, Expert Group on Seals (2008). Status of Stocks.

[52] Payne M.R. (1978). Population size and age determination in the Antarctic fur seal *Arctocephalus gazella*. Mammal Rev..

[53] Bonner W.N., Siegfried W.R., Condy P.R., Laws R.M. (1985). Impact of fur seals on the terrestrial environment at South Georgia. Antarctic Nutrient Cycles and Food Webs.

[54] Croxall J.P., McCann T.S., Prince P.A., Rothery P., Sahrhage D. (1988). Reproductive performance of seabirds and seals at South Georgia and Signey Island, South Orkney Islands, 1976–1987: Implications for Southern Ocean monitoring studies. Antarctic Ocean and Resources Variability.

[55] Foley C.M., Lynch H.J. (2020). A method to estimate pre-exploitation population size. Conserv. Biol..

[56] Laws R.M. (1973). Population increase of fur seals at South Georgia. Polar Rec..

[57] Bonner W.N. The status of the Antarctic fur seal *Arctocephalus gazella*. Proceedings of the Advisory Committee on Marine Resources Research, Scientific Consultation on Marine Mammals.

[58] Leaper R., Bannister J.L., Branch T.A., Clapham P., Donovan G., Matsuoka K., Reilly S., Zerbini A. (2008). A Review of Abundance, Trends and Foraging Parameters of Baleen Whales in the Southern Hemisphere.

[59] Hoffman J.I., Grant S.M., Forcada J., Phillips C.D. (2011). Bayesian inference of a historical bottleneck in a heavily exploited marine mammal. Mol. Ecol..

[60] Humble E., Dasmahapatra K.K., Martinez-Barrio A., Gregorio I., Forcada J., Polikeit A.C., Goldsworthy S.D., Goebel M.E., Kalinowski J., Wolf J.B.W. (2018). RAD Sequencing and a hybrid Antarctic fur seal genome assembly reveal rapidly decaying linkage disequilibrium, global population structure and evidence for inbreeding. G3 Genes Genomes Genet..

[61] Nagel R., Kaiser S., Stainfield C., Toscani C., Fox-Clarke C., Paijmans A.J., Costa Castro C., Vendrami D., Forcada J., Hoffman J.I. (2021). Low heritability and high phenotypic plasticity of cortisol in response to environmental heterogeneity in fur seals. bioRxiv.

[62] Etter P.D., Preston J.L., Bassham S., Cresko W.A., Johnson E.A. (2011). Local de novo assembly of RAD paired-end contigs using short sequencing reads. PLoS ONE.

[63] Catchen J.M., Amores A., Hohenlohe P., Cresko W., Postlethwait J.H. (2011). Stacks: Building and genotyping Loci de novo from short-read sequences. G3 Genes Genomes Genet..

[64] Peart C.R., Williams C., Pophaly S.D., Johnson J., Neely B.A., Gulland F., Adams D., Ng B.L., Cheng W., Goebel M.E. (2021). Hi-C scaffolded short- and long-read genome assemblies of the California sea lion are broadly consistent for syntenic inference across 45 million years of evolution. Mol. Ecol. Resour..

[65] Li H. (2013). Aligning sequence reads, clone sequences and assembly contigs with BWA-MEM. arXiv.

[66] Li H. (2011). A statistical framework for SNP calling, mutation discovery, association mapping and population genetical parameter estimation from sequencing data. Bioinformatics.

[67] Korneliussen T.S., Albrechtsen A., Nielsen R. (2014). ANGSD: Analysis of Next Generation Sequencing Data. BMC Bioinform..

[68] Forcada J., Trathan P.N., Murphy E.J. (2008). Life history buffering in Antarctic mammals and birds against changing patterns of climate and environmental variation. Glob. Change Biol..

[69] Dornburg A., Brandley M.C., McGowen M.R., Near T.J. (2012). Relaxed Clocks and Inferences of Heterogeneous Patterns of Nucleotide Substitution and Divergence Time Estimates across Whales and Dolphins (Mammalia: Cetacea). Mol. Biol. Evol..

[70] Li H., Durbin R. (2011). Inference of human population history from individual whole-genome sequences. Nature.

[71] Stoffel M.A., Humble E., Paijmans A.J., Acevedo-Whitehouse K., Chilvers B.L., Dickerson B., Galimberti F., Gemmell N.J., Goldsworthy S.D., Nichols H.J. (2018). Demographic histories and genetic diversity across pinnipeds are shaped by human exploitation, ecology and life-history. Nat. Commun..

[72] Paijmans A.J., Stoffel M.A., Bester M.N., Cleary A.C., De Bruyn P.J.N., Forcada J., Goebel M.E., Goldsworthy S.D., Guinet C., Lydersen C. (2020). The genetic legacy of extreme exploitation in a polar vertebrate. Sci. Rep..

[73] Cleary A.C., Bester M., Forcada J., Goebel M., Goldsworthy S.D., Guinet C., Hoffman J.I., Kovacs K.M., Lydersen C., Lowther A.D. (2019). Prey differences drive local genetic adaptation in Antarctic fur seals. Mar. Ecol. Prog. Ser..

[74] Hoffman J.I., Bauer E., Paijmans A.J., Humble E., Beckmann L.M., Kubetschek C., Christaller F., Krocker N., Fuchs B., Moreras A. (2018). A global cline in a colour polymorphism suggests a limited contribution of gene flow towards the recovery of a heavily exploited marine mammal. R. Soc. Open Sci..

[75] Wynen L.P., Goldsworthy S.D., Guinet C., Bester M.N., Boyd I.L., Gjertz I., Hofmeyr G.J., White R.W., Slade R. (2000). Postsealing genetic variation and population structure of two species of fur seal (*Arctocephalus gazella* and *A. tropicalis*). Mol. Ecol..

[76] Palstra F.P., Ruzzante D.E. (2008). Genetic estimates of contemporary effective population size: What can they tell us about the importance of genetic stochasticity for wild population persistence?. Mol. Ecol..

[77] Nagel R., Stainfield C., Fox-Clarke C., Toscani C., Forcada J., Hoffman J.I. (2021). Evidence for an Allee effect in a declining fur seal population. Proc. R. Soc. B Biol. Sci..

[78] Han E., Sinsheimer J.S., Novembre J. (2014). Characterizing Bias in Population Genetic Inferences from Low-Coverage Sequencing Data. Mol. Biol. Evol..

[79] Fountain E.D., Pauli J.N., Reid B.N., Palsbøll P.J., Peery M.Z. (2016). Finding the right coverage: The impact of coverage and sequence quality on single nucleotide polymorphism genotyping error rates. Mol. Ecol. Resour..

[80] Shafer A.B.A., Peart C.R., Tusso S., Maayan I., Brelsford A., Wheat C.W., Wolf J.B.W. (2017). Bioinformatic processing of RAD-seq data dramatically impacts downstream population genetic inference. Methods Ecol. Evol..

[81] Le S.Q., Durbin R. (2011). SNP detection and genotyping from low-coverage sequencing data on multiple diploid samples. Genome Res..

[82] Myers S., Fefferman C., Patterson N. (2008). Can one learn history from the allelic spectrum?. Theor. Popul. Biol..

[83] Mill H.R. (1905). The Seige of the South Pole: The Story of Antarctic Exploration.

[84] Fanning E. (1924). Voyages and Discoveries in the South Seas 1792–1832.

