# Supplementary material for: Demographic Reconstruction of Antarctic Fur Seals Supports the Krill Surplus Hypothesis
Source: Genes (Basel). 2022 Mar 18;13(3):541. doi: 10.3390/genes13030541 (PMC8954904; doi:10.3390/genes13030541)
Supplement: Supplementary file 1 [file genes-13-00541-s001.zip › genes-1620491-supplementary.pdf]

Supplementary Figure S1 from Demographic reconstruction of Antarctic fur seals supports the krill surplus hypothesis

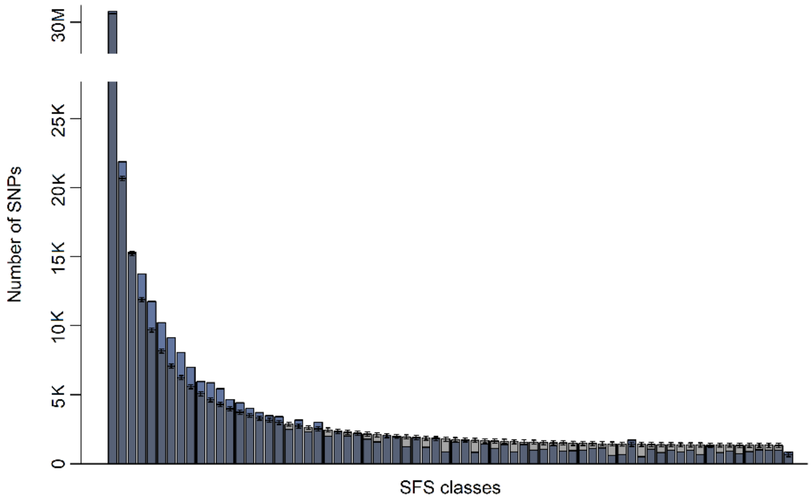

Figure S1: Observed and simulated site frequency spectra (SFS). The blue bars represent the empirical SFS and the grey bars represent the mean SFS across 600 simulations based on the maximum likelihood parameter estimates from the best supported model. The dark grey lines represent the 95% CIs of these simulations. The first bar represents the number of monomorphic sites and the second bar represents the number of doubletons. Singletons were excluded from the analysis as described in the Materials and methods.
